# Supplementary material for: Pooled Analysis of the Effect of Pre-Existing Ad5 Neutralizing Antibodies on the Immunogenicity of Adenovirus Type 5 Vector-Based COVID-19 Vaccine from Eight Clinical Trials
Source: Vaccines (Basel). 2025 Mar 20;13(3):333. doi: 10.3390/vaccines13030333 (PMC11945733; doi:10.3390/vaccines13030333)
Supplement: Supplementary file 1 [file vaccines-13-00333-s001.zip › vaccines-3522770-supplementary.pdf]

Table S1. Clinical trials included in this pooled analysis.

NO.	Trials[a] (ClinicalTrials.gov Identifier)	Title[b]	Age range	Immunization schedule [dosage]	Sample size (PPS)
1	NCT04313127	Phase I Clinical Trial of a COVId-19 Vaccine in 18-60 Healthy Adults (CTCOVId-19)	18-60 years	Ad5-IM [5.0×10^{10}vp]	35
				Ad5-IM [1.0×10^{11}vp]	35
				Ad5-IM [1.5×10^{11}vp]	36
2	NCT04341389	A Phase II Clinical Trial to Evaluate the Recombinant Vaccine for COVId-19 (Adenovirus Vector) (CTII-nCoV)	18 years or older	Ad5-IM [5.0×10^{10}vp]	124
				Ad5-IM [1.0×10^{11}vp]	243
3	NCT04916886	A Clinical Trial of Immunobridging and Lot-to-lot Consistency of COVId-19 Vaccine (Ad5-nCoV) in different Age Groups	6-17 years	Ad5-IM [3.0×10^{10}vp]	1001
			18-59 years	Ad5-IM [1.0×10^{11}vp]	977
4	NCT04892459	Study on Sequential Immunization of Inactivated SARS-CoV-2 Vaccine and Recombinant SARS-CoV-2 Vaccine (Ad5 Vector)	18-59 years	ICV×1+Ad5-IM [5×10^{10}vp]	90
				ICV×2+Ad5-IM [5×10^{10}vp]	46
5	NCT04952727	Study on Sequential Immunization of Inactivated COVId-19 Vaccine and Recombinant COVId-19 Vaccine (Ad5 Vector) in Elderly Adults	60 years or older	ICV×1+Ad5-IM [5×10^{10}vp]	47
				ICV×2+Ad5-IM [5×10^{10}vp]	96
6	NCT05330871	Evaluate the Safety and Immunogenicity of Ad5 COVId-19 Vaccines for Booster Use in Children Aged 6-17 Years	6-17 years	ICV×2+Ad5-IM [3×10^{10}vp]	65
				ICV×2+Ad5-IH [1×10^{10}vp]	207
7	NCT05043259	Heterologous Prime-boost Immunization With an Aerosolised Adenovirus Type-5 Vector-based COVId-19 Vaccine (Ad5-nCoV) After Priming With an Inactivated SARS-CoV-2 Vaccine	18 years or older	ICV×2+Ad5-IH [1×10^{10}vp]	138
				ICV×2+Ad5-IH [2×10^{10}vp]	139
8	NCT05303584	Heterologous Boost Immunization With Ad5-nCoV After Three-dose Priming With an Inactivated SARS-CoV-2 Vaccine	18 years or older	ICV×3+Ad5-IM [5×10^{10}vp]	117
				ICV×3+Ad5-IH [1×10^{10}vp]	116

[a]All trials in this study are registered with ClinicalTrials.gov. The numbers starting with the characters NCT_ indicates ClinicalTrials.gov Identifier. [b]The title listed in the table is consistent with ClinicalTrials.gov registration name. Abbreviation: vp=viral particles; Ad5-IM= administering Ad5-nCoV by intramuscular injection; Ad5-IH= administering Ad5-nCoV by aerosolized inhalation; ICV= administering inactivated COVID-19 vaccine by intramuscular injection.

Table S2. Neutralizing antibodies against live SARS-CoV-2 at day 28 post-vaccination in the adolescents cohort aged 6-17 years and the adults cohort aged 18 years and older.

	Adolescents cohort aged 6-17 years (N=1273)				Adults cohort aged 18 years and older (N=2239)			
	**Ad5-IM-prime**	**Ad5-IM-boost**	**Ad5-IH-boost**	***P value**	**Ad5-IM-prime**	**Ad5-IM-boost**	**Ad5-IH-boost**	***P value**
**Total population**								
**n**	1001	65	207		1450	396	393	
**GMT**	18.5 (17.3, 19.9)	349.3 (290.8, 419.5)	461.1 (400.9, 530.3)	0.1676	13.9 (13.1, 14.7)	194.5 (172.0, 219.9)	1102.7 (982.6, 1237.5)	<0.0001
**GMFI**	9.3 (8.6, 9.9)	83.5 (69.7, 100.1)	102.8 (88.9, 118.8)	0.3723	5.5 (5.2, 5.9)	52.2 (46.4, 58.8)	210.8 (180.0, 246.9)	<0.0001
**Seroconversion**	863 (86.2%)	65 (100.0%)	203 (98.1%)	0.5755	977 (67.4%)	394 (99.5%)	390 (99.2%)	0.6475
**Participants with pre-existing Ad5 NAb titers < 1:12**								
**n**	336	32	76		297	66	59	
**GMT**	36.5 (33.0, 40.4)	428.4 (329.8, 556.6)	819.2 (698.8, 960.3)	0.0016	35.6 (32.0, 39.7)	358.3 (267.6, 479.6)	2414.1 (2006.9, 2904.0)	<0.0001
**GMFI**	18.3 (16.5, 20.2)	103.5 (80.0, 133.9)	186.9 (158.4, 220.6)	0.0049	12.9 (11.5, 14.3)	92.4 (72.0, 118.6)	414.4 (286.5, 599.4)	<0.0001
**1:12 ≤ Participants with pre-existing Ad5 NAb titers ≤ 1:200**								
**n**	114	7	21		364	109	94	
**GMT**	25.4 (21.9, 29.4)	336.3 (162.1, 697.6)	499.7 (375.6, 664.8)	0.4678	14.1 (12.6, 15.6)	191.1 (148.7, 245.6)	1458.9 (1182.5, 1799.8)	<0.0001
**GMFI**	12.7 (11.0, 14.7)	84.1 (46.9, 150.8)	105.3 (74.1, 149.8)	0.7933	6.0 (5.4, 6.7)	58.2 (45.5,74.3)	314.7 (240.0, 412.6)	<0.0001
**1:200 < Participants with pre-existing Ad5 NAb titers ≤ 1:1000**								
**n**	425	13	69		593	175	160	
**GMT**	12.0 (10.9, 13.2)	315.6 (211.9, 470.0)	368.7 (284.3, 478.0)	0.8712	9.4 (8.6, 10.2)	178.5 (149.9, 212.6)	842.5 (695.4, 1020.8)	<0.0001
**GMFI**	6.0 (5.4, 6.6)	68.7 (48.5, 97.3)	83.1 (64.4, 107.2)	0.8148	4.1 (3.8, 4.5)	44.8 (37.8, 53.1)	145.7 (111.8, 189.9)	0.0069
**Participants with pre-existing Ad5 NAb titers > 1:1000**								
**n**	126	13	41		196	46	80	
**GMT**	9.9 (8.3, 11.9)	238.6 (153.7, 370.5)	222.2 (161.7, 305.2)	0.9725	10.7 (9.1, 12.6)	116.9 (84.9, 161.1)	762.7 (596.2, 975.8)	<0.0001

**GMFI**	5.0 (4.2, 5.9)	59.6 (40.2, 88.6)	47.9 (34.7, 66.1)	0.7726	3.2 (2.8, 3.8)	32.0 (23.3, 44.0)	167.4 (120.4, 232.9)	<0.0001

Data are mean (95%CI) or n, %. n = number of participants. % = proportion of participants. GMT= geometric mean antibody titer. GMFI=geometric mean fold increase. Seroconversion was defined as at least a 4-times increase in the antibody titre 28 days after vaccination. *The p-value indicates the statistical difference between the Ad5-IM-boost subgroup and the Ad5-IH-boost subgroup, as both of these subgroups show a significant statistical difference (p<0.0001) when compared to the Ad5-IM-prime subgroup.

Table S3. Multivariate linear regression analysis of the participants receiving one shot of Ad5-nCoV by intramuscular injection as a primary immunization strategy.

**Variable**		**Model 1**		**Model 2**	
		**Estimate**	**P value**	**Estimate**	**P value**
**Ad5-IM-prime subgroup in the adolescents cohort (n=1001)**					
Intercept		67.6	<0.0001	100.0	<0.0001
Age		-1.0	<0.0001	-1.0	<0.0001
Sex	Male	Reference	Reference	Reference	Reference
	Female	-1.1	0.0373	-1.1	0.0350
[*]Pre-existing Ad5 NAb titers		——	——	-1.6	<0.0001
[†]Pre-existing Ad5 NAb titers	negative (less than 1:12)	Reference	Reference	——	——
	low (1:12 to 1:200)	-1.4	0.0009	——	——
	moderate (1:200 to 1:1,000)	-2.9	<0.0001	——	——
	high (greater than 1:1,000)	-3.5	<0.0001	——	——
**Ad5-IM-prime subgroup in the adults cohort (n=1450)**					
Intercept		125.9	<0.0001	199.5	<0.0001

Age		-1.0	<0.0001	-1.0	<0.0001
Sex	Male	Reference	Reference	Reference	Reference
	Female	1.1	0.2269	1.1	0.2028
Dosage	1.5×10^{11}vp	Reference	Reference	Reference	Reference
	1.0×10^{11}vp	-1.5	0.0133	-1.6	0.0104
	5.0×10^{10}vp	-2.0	<0.0001	-2.2	<0.0001
*Pre-existing Ad5 NAb titers		——	——	-1.7	<0.0001
†Pre-existing Ad5 NAb titers	negative (less than 1:12)	Reference	Reference	——	——
	low (1:12 to 1:200)	-2.1	<0.0001	——	——
	moderate (1:200 to 1:1,000)	-3.4	<0.0001	——	——
	high (greater than 1:1,000)	-3.5	<0.0001	——	——

*Pre-existing Ad5 NAbs are continuous variables. † Pre-existing Ad5 NAbs are categorical variables. vp=viral particles.

Table S4. Multivariate linear regression analysis of the participants receiving one shot of Ad5-nCoV by intramuscular injection or aerosolized inhalation as a heterologous sequential boosting immunization strategy.

Variable	Model 1		Model 2	
	Estimate	P value	Estimate	P value
**Ad5-IM-boost and Ad5-IH-boost subgroup in the adolescents cohort (n=272)**				
Intercept	223.9	<0.0001	316.2	<0.0001
Age	1.0	0.6254	1.0	0.7406

Sex	Male	Reference	Reference	Reference	Reference
	Female	-1.0	0.6702	-1.0	0.6616
Day 0 neutralising antibodies to live SARS-CoV-2, titers		1.8	0.1328	1.7	0.1761
Time since the last dose of inactivated vaccine, days		1.0	0.5388	1.0	0.4953
Subgroup	Ad5-IM-boost	Reference	Reference	Reference	Reference
	Ad5-IH-boost	1.4	0.0066	1.4	0.0071
[*]Pre-existing Ad5 NAb titers		——	——	-1.5	<0.0001
[†]Pre-existing Ad5 NAb titers	negative (less than 1:12)	Reference	Reference	——	——
	low (1:12 to 1:200)	-1.5	0.0168	——	——
	moderate (1:200 to 1:1,000)	-2.0	<0.0001	——	——
	high (greater than 1:1,000)	-3.2	<0.0001	——	——
**Ad5-IM-boost and Ad5-IH-boost subgroup in the adults cohort (n=789)**					
Intercept		288.4	<0.0001	371.5	<0.0001
Age		-1.0	0.0003	-1.0	<0.0001
Sex	Male	Reference	Reference	Reference	Reference
	Female	-1.1	0.4009	-1.1	0.4320
Day 0 neutralising antibodies to live SARS-CoV-2		1.2	0.0714	1.2	0.0973
Time since the last dose of inactivated vaccine,days		1.0	<0.0001	1.0	<0.0001

Subgroup	Ad5-IM-boost	Reference	Reference	Reference	Reference
	Ad5-IH-boost	3.9	<0.0001	3.9	<0.0001
*Pre-existing Ad5 NAb titers		——	——	-1.4	<0.0001
†Pre-existing Ad5 NAb titers	negative (less than 1:12)	Reference	Reference	——	——
	low (1:12 to 1:200)	-1.7	<0.0001	——	——
	moderate (1:200 to 1:1,000)	-2.4	<0.0001	——	——
	high (greater than 1:1,000)	-3.0	<0.0001	——	——

*Pre-existing Ad5 NAbs are continuous variables. † Pre-existing Ad5 NAbs are categorical variables. vp=viral particles.
